# H2V: a database of human genes and proteins that respond to SARS-CoV-2, SARS-CoV, and MERS-CoV infection

**DOI:** 10.1186/s12859-020-03935-2

**Published:** 2021-01-07

**Authors:** Nan Zhou, Jinku Bao, Yuping Ning

**Affiliations:** 1 Affiliated Brain Hospital of Guangzhou Medical University, 36 Mingxin Rd, Guangzhou, 510370 China; 2 Guangzhou Huiai Hospital, 36 Mingxin Rd, Guangzhou, 510370 China; 3 Guangdong Engineering Technology Research Center for Translational Medicine of Mental Disorders, 36 Mingxin Rd, Guangzhou, 510370 China; 4 College of Life Sciences, Sichuan University, 29 Wangjiang Rd, Chengdu, 610064 China

**Keywords:** Database, Coronavirus, Protein, Gene

## Abstract

**Background:**

The ongoing global COVID-19 pandemic is caused by SARS-CoV-2, a novel coronavirus first discovered at the end of 2019. It has led to more than 50 million confirmed cases and more than 1 million deaths across 219 countries as of 11 November 2020, according to WHO statistics. SARS-CoV-2, SARS-CoV, and MERS-CoV are similar. They are highly pathogenic and threaten public health, impair the economy, and inflict long-term impacts on society. No drug or vaccine has been approved as a treatment for these viruses. Efforts to develop antiviral measures have been hampered by the insufficient understanding of how the human body responds to viral infections at the cellular and molecular levels.

**Results:**

In this study, journal articles and transcriptomic and proteomic data surveying coronavirus infections were collected. Response genes and proteins were then identified by differential analyses comparing gene/protein levels between infected and control samples. Finally, the H2V database was created to contain the human genes and proteins that respond to SARS-CoV-2, SARS-CoV, and MERS-CoV infection.

**Conclusions:**

H2V provides molecular information about the human response to infection. It can be a powerful tool to discover cellular pathways and processes relevant for viral pathogenesis to identify potential drug targets. It is expected to accelerate the process of antiviral agent development and to inform preparations for potential future coronavirus-related emergencies. The database is available at: http://www.zhounan.org/h2v.

## Background

Coronaviruses are single-stranded RNA viruses, and some can cross species barriers to cause deadly and infectious respiratory disease in humans [1]. A novel coronavirus that causes viral pneumonia was reported in December 2019 [2]. The virus, now known as SARS-CoV-2, is commonly asymptomatic and contagious prior to symptom onset [3]. These characteristics contribute to the difficulty in containing the virus. As a result, SARS-CoV-2 spread rapidly worldwide and caused the ongoing COVID-19 pandemic.

The two most recent coronavirus disease epidemics were severe acute respiratory syndrome (SARS) in 2002–2003 and Middle East respiratory syndrome (MERS) in 2012 [4]. With a case fatality rate of ~ 10%, SARS-related coronavirus (SARS-CoV) infected 8098 people and caused 774 deaths; MERS-related coronavirus (MERS-CoV) has a higher mortality rate of ~ 34%, and it has resulted in ~ 2500 confirmed cases and ~ 900 deaths to date [5]. The average case fatality rate of COVID-19 is ~ 2%, though the risk of serious complications and mortality increases dramatically at later ages [6]. The mortality rate is < 0.1% in children but increases to 10% or higher in older people [7]. In terms of the absolute number of cases and deaths, the COVID-19 pandemic is more severe than the previous two outbreaks. As of 11 November 2020, > 50 million confirmed cases and > 1 million deaths have been reported to the WHO (https://www.who.int) worldwide. It is urgent for the world to unite to find effective ways to bring the COVID-19 crisis to an end.

SARS-CoV-2, SARS-CoV and MERS-CoV are beta-coronaviruses that can cause serious health consequences in humans. Two other beta-coronaviruses, HCoV-OC43 and HKU1, can also infect humans but only cause self-limiting flu-like illness [8]. Even though the world has repeatedly suffered from coronavirus outbreaks, there are no clinically effective prophylactics or therapeutics available. The clinical management of COVID-19, as well as SARS and MERS, is largely limited to infection prevention and supportive care. This highlights the need to develop therapies to treat coronavirus-related diseases.

The life cycle of coronavirus includes several key steps: viral entry, genomic RNA replication, mRNA translation, protein processing, and virion assembly and release [9]. The interplay between host cells and viruses at the viral entry stage has been well documented. To enter human cells, both SARS-CoV-2 and SARS-CoV bind via their S proteins to the cell surface receptor angiotensin-converting enzyme 2 (ACE2) [10]. MERS-CoV enters the human cell by binding another receptor, dipeptidyl peptidase 4 (DPP4) [4]. Hoffmann and colleagues have also proven that the binding of the SARS-CoV-2 S protein to human ACE2 additionally depends on TMPRSS2 and have shown that cellular entry of SARS-CoV-2 can be blocked by the serine protease inhibitor camostat mesylate [11]. More details about the interplay between humans and viruses at other viral life cycle stages remain to be elucidated. There is no doubt that the human body responds to viral infection, and the response can be detected at the molecular level by genome- and proteome-wide measurements.

Although SARS-CoV suddenly disappeared in the summer of 2003, MERS-CoV is occasionally observed, and SARS-CoV-2 continues to spread rapidly in some parts of the world. The spread of SARS-CoV-2 has worsened to the extent that the winter 2020 wave of COVID-19 has forced new lockdowns in some European cities. For normal life to resume, specific drugs against COVID-19 are urgently required but remain unavailable. Additionally, there is no cure for SARS and MERS, indicating that our understanding of these dangerous coronaviruses is very limited. Given that knowledge of cellular responses to viral infections is essential for establishing therapeutics, we identified human proteins and genes that respond to SARS-CoV-2, SARS-CoV and MERS-CoV infections and subsequently developed the H2V database in the present study.

## Construction and content

### Data collection

In this study, human proteins/genes responding to viral infections were defined as differentially expressed genes (DEGs), proteins that participate in human-virus protein–protein interactions (PPIs), differentially expressed proteins (DEPs), differentially phosphorylated proteins (DPPs), differentially translated proteins (DTPs), differentially ubiquitinated proteins (DUPs), and disease severity associated proteins (SAPs).

We used the Bing search engine (https://www.bing.com), NCBI resources (https://www.ncbi.nlm.nih.gov/), and Proteome Xchange database http://www.proteomexchange.org/) to search for studies of SARS-CoV-2, SARS-CoV, and MERS-CoV infection. Based on the definition of response genes/proteins, the studies were classified as DEG, PPI, DEP, DPP, DTP, DUP and SAP. For each study type, three independent studies per virus were selected. If the number of available studies was less than three, any identified sources were used. Since we focused on dynamic changes in response genes/proteins over time post infection, studies reporting time-course surveys were selected as the highest priority. Only in cases of insufficient study numbers were studies without time-course examinations selected. After study selection, the journal articles reporting the selected studies were retrieved, and information about gene and protein responses was extracted from the main text and supplementary material of each article. When such information was not available in the journal article, raw data from the selected studies were downloaded from public repositories and subsequently analyzed. The selected studies ( [12–24]) and corresponding strategies to identify response genes and proteins are summarized in Table 1.

**Table 1:** Studies and strategies used to identify response genes/proteins

Study^1^	Type	Strategy
*SARS-CoV-2*		
SRA: SRP257667	DEG	b
https://doi.org/10.1101/2020.06.17.156455	DEG	a
PMID: 32358202	DEG	c
PMID: 32353859	PPI	a
https://doi.org/10.1101/2020.06.17.156455	PPI	a
PMID: 33060197	PPI	a
PMID: 32408336	DEP	a
https://doi.org/10.1101/2020.06.17.156455	DEP	a
PMID: 32645325	DPP	a
https://doi.org/10.1101/2020.06.17.156455	DPP	a
PMID: 32877642	DPP	a
PMID: 32408336	DTP	a
https://doi.org/10.1101/2020.06.17.156455	DUP	a
PMID: 32492406	SAP	a
https://doi.org/10.1101/2020.05.02.20088666	SAP	a
*SARS-CoV*		
PMID: 32358202	DEG	c
PMID: 23935999	DEG	d
PMID: 20090954	DEG	d
PMID: 33060197	PPI	a
https://doi.org/10.1101/2020.06.17.156455	PPI	a
PMID: 15784933	DEP	a
*MERS-CoV*		
PMID: 32223537	DEG	a
GEO: GSE81909	DEG	d
GEO: GSE79458	DEG	d
PMID: 33060197	PPI	a

^1^If a PMID was not available, an alternative database accession number is used

a: Response genes/proteins were extracted from the journal article

b: Response genes/proteins were identified from RNA-seq data using RaNA-seq, with p < 0.05 and |log2(fold change)|> 1 at any timepoint post infection

c: Response genes/proteins were identified from read counts from GEO using DESeq2, with p < 0.05 and |log2(fold change)|> 1 at any timepoint post infection

d: Response genes/proteins were identified from the expression matrix from GEO using limma, with p < 0.05 and |log2(fold change)|> 1 at any timepoint post infection

Genome assemblies MN985325.1, NC_004718.3 and NC_019843.3 from the NCBI database (https://www.ncbi.nlm.nih.gov/) were used to annotate SARS-CoV-2, SARS-CoV and MERS-CoV genes, respectively. Drug information was collected from the DrugBank database [25]. Postprocessing of data was performed using R (https://www.r-project.org/) and Python (https://python.org/).

### Implementation

H2V was developed using conventional web development techniques. The user interface was developed using HTML5, CSS3, and JavaScript. Bootstrap v4 (https://getbootstrap.com/) was used for layout design. DataTables (https://datatables.net/) was used to organize data in tables on the web page. Cytoscape.js was used for network visualization [26]. Plotly (https://plotly.com/) was used to create interactive plots. PHP (https://www.php.net/), Python (https://www.python.org) and Bash scripts were used for server-side development. The SQLite (https://www.sqlite.org/) database was used to manage the data. NCBI’s sequence viewer (https://www.ncbi.nlm.nih.gov/projects/sviewer/) was embedded on the web page to show the viral genome. PANTHER API was used for pathway enrichment analysis [27]. Drug information is not stored in H2V; instead, it is automatically retrieved on request from the DrugBank database via UniProt’s REST API [28]. H2V is deployed in an Amazon AWS host running Ubuntu 16.04.

## Utility and discussion

### Statistics of H2V data

Due to the variation in the availability of studies, the H2V datasets vary among the three viruses. As shown in Table 2, seven datasets of genes/proteins that respond to SARS-CoV-2 infection are available, namely, DEGs, PPIs, DEPs, DPPs, DTPs, DUPs and SAPs. In comparison, only three (DEGs, PPIs and DEPs) and two (DEGs and PPIs) datasets of genes/proteins that respond to SARS-CoV and MERS-CoV infections, respectively, are available. DEGs datasets are available for the response to infections with all three viruses. A total of 9321 human genes responded to MERS-CoV infection, while fewer genes (2249) responded to SARS-CoV infection and even fewer (1395) to SARS-CoV-2 infection. PPIs datasets are also available for the response to infections with all three viruses. There are 1581, 1150, and 296 interaction pairs of human and corresponding SARS-CoV-2, SARS-CoV and MERS-CoV proteins. DEPs datasets are available for the response to SARS-CoV-2 and SARS-CoV infections and include 253 and 66 human proteins, respectively, that responded to the infections. DPPs, DTPs, DUPs and SAPs datasets are only available for the response to SARS-CoV-2 infection, and include 2198 (5046 phosphorylation sites), 232, 516 (730 ubiquitination sites) and 610 response proteins, respectively.

**Table2:** Statistics of data in H2V

Virus	Dataset	Number of entries
SARS-CoV-2	DEGs	1395
	PPIs	1581
	DEPs	253
	DPPs	5046
	DTPs	232
	DUPs	730
	SAPs	610
SARS-CoV	DEGs	2249
	PPIs	1150
	DEPs	66
MERS-CoV	DEGs	9321
	PPIs	296

**Fig. 1:**
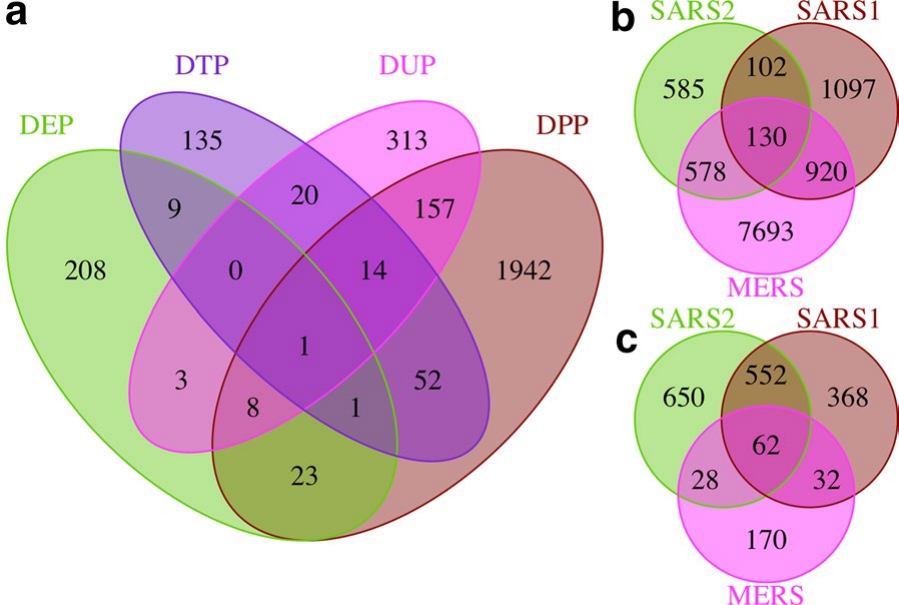
Venn diagrams showing intersections of response genes/proteins. **a** The intersection between DEPs, DPPs, DTPs, and DUPs in response to SARS-CoV-2 infection. **b** The intersection between DEGs in response to SARS-CoV-2, SARS-CoV, and MERS-CoV infection. **c** The intersection between PPIs in response to SARS-CoV-2, SARS-CoV, and MERS-CoV infection. SARS2 denotes SARS-CoV-2; SARS1 denotes SARS-CoV; MERS denotes MERS-CoV

To determine whether common proteins participate in different processes in response to SARS-CoV-2 infection, the intersection of DEPs, DPPs, DTPs and DUPs was analyzed. Figure 1a shows that both expression and translation of 11 proteins changed dramatically upon infection, that both phosphorylation and ubiquitination of 180 proteins changed remarkably upon infection and that one protein underwent noticeable changes in expression, phosphorylation, translation and ubiquitination. We then used Venn diagrams to analyze genes/proteins that are common across responses to different viral infections. This would help to elucidate the fundamental mechanisms of viral pathogenesis. Figure 1b shows that 130 common genes exhibited significant differences in expression upon infection. Figure 1c shows that 62 human proteins could interact with all three viruses.

### Overview of H2V

**Fig. 2:**
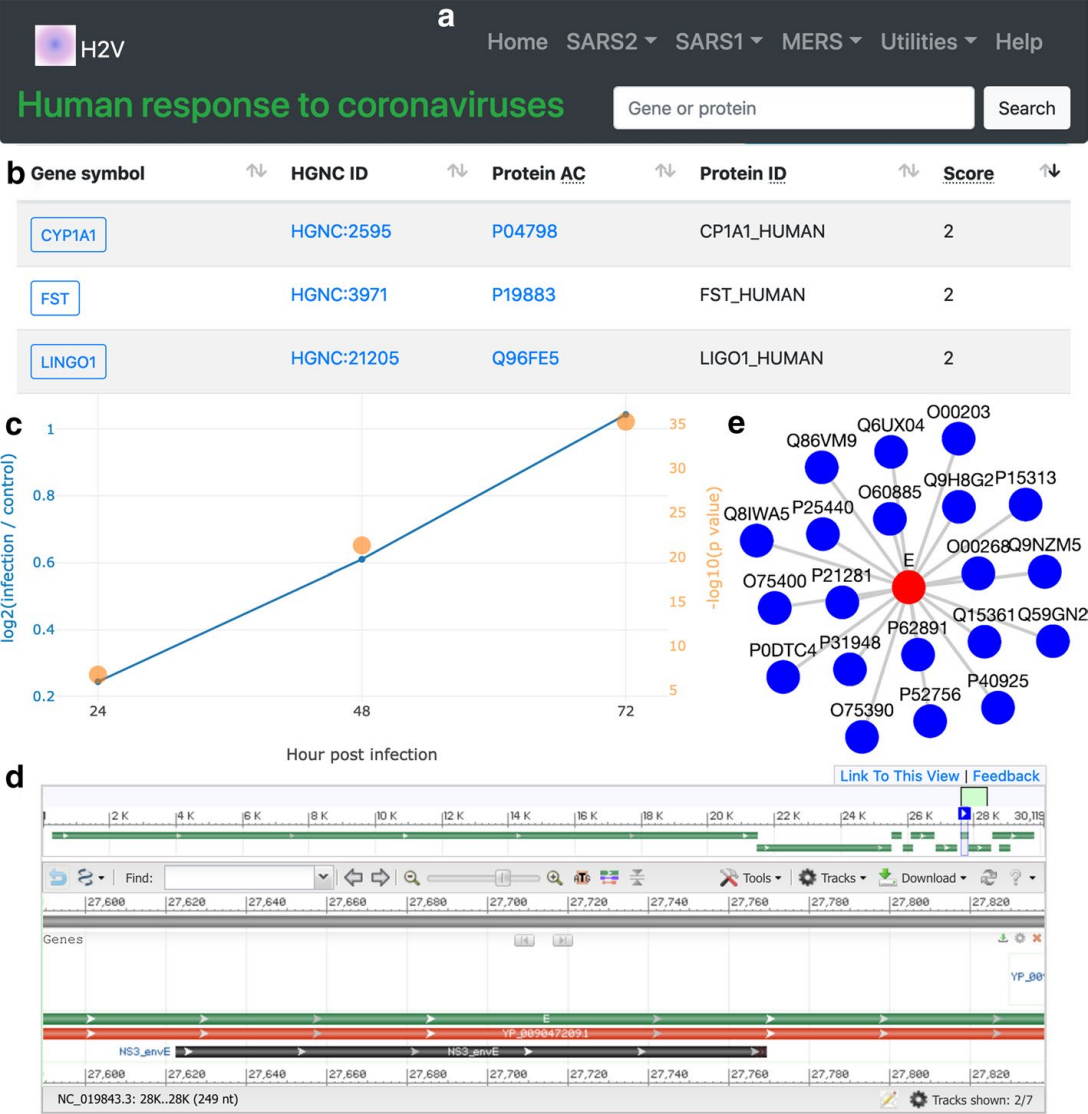
H2V overview. **a** Header. **b** An example data table. **c** An example temporal profile. The x-axis shows the timepoints, the left y-axis shows the log2-transformed fold change, and the right y-axis shows the negation of the log10-transformed p value. **d** The embedded NCBI sequence viewer. **e** An example visualization of a subset of PPIs. Red nodes denote viral proteins; blue nodes denote human proteins

As shown in Fig. 2a, the web page header contains a navigation bar and a search box. The search box accepts queries from the user and tries to match anything that resembles a gene or protein. The navigation bar provides access to all resources in the database. The “SARS2” drop-down menu is linked to the SARS-CoV-2 infection response genes/proteins. Similarly, the “SARS1” and “MERS” drop-down menus link to the SARS-CoV-1 and MERS-CoV infection response genes/proteins, respectively. Under the “Utilities” drop-down menu, useful utilities, including a link to download data from or upload data to H2V, are provided. On the page listing the response genes/proteins, the genes/proteins are shown within rows of a table, with additional information about the gene/protein shown in columns (Fig. 2b). The “Score” column in the table indicates the reliability of the gene/protein, calculated as the number of studies in which the gene/protein was identified [29]. The genes/proteins in the table are clickable. Clicking on a gene/protein will link to another page showing details of how the gene/protein responds to viral infection. This page includes two helpful features: one is to examine changes in the gene/protein at different timepoints post infection (Fig. 2c), and the other is to discover known drugs that target the gene/protein. For PPIs, an embedded sequence viewer, as shown in Fig. 2d, is provided for easy inspection of the gene/protein annotation in the viral genome. In addition, PPIs can also be visualized as an interaction network on the page (Fig. 2e).

### Application cases

**Fig. 3:**
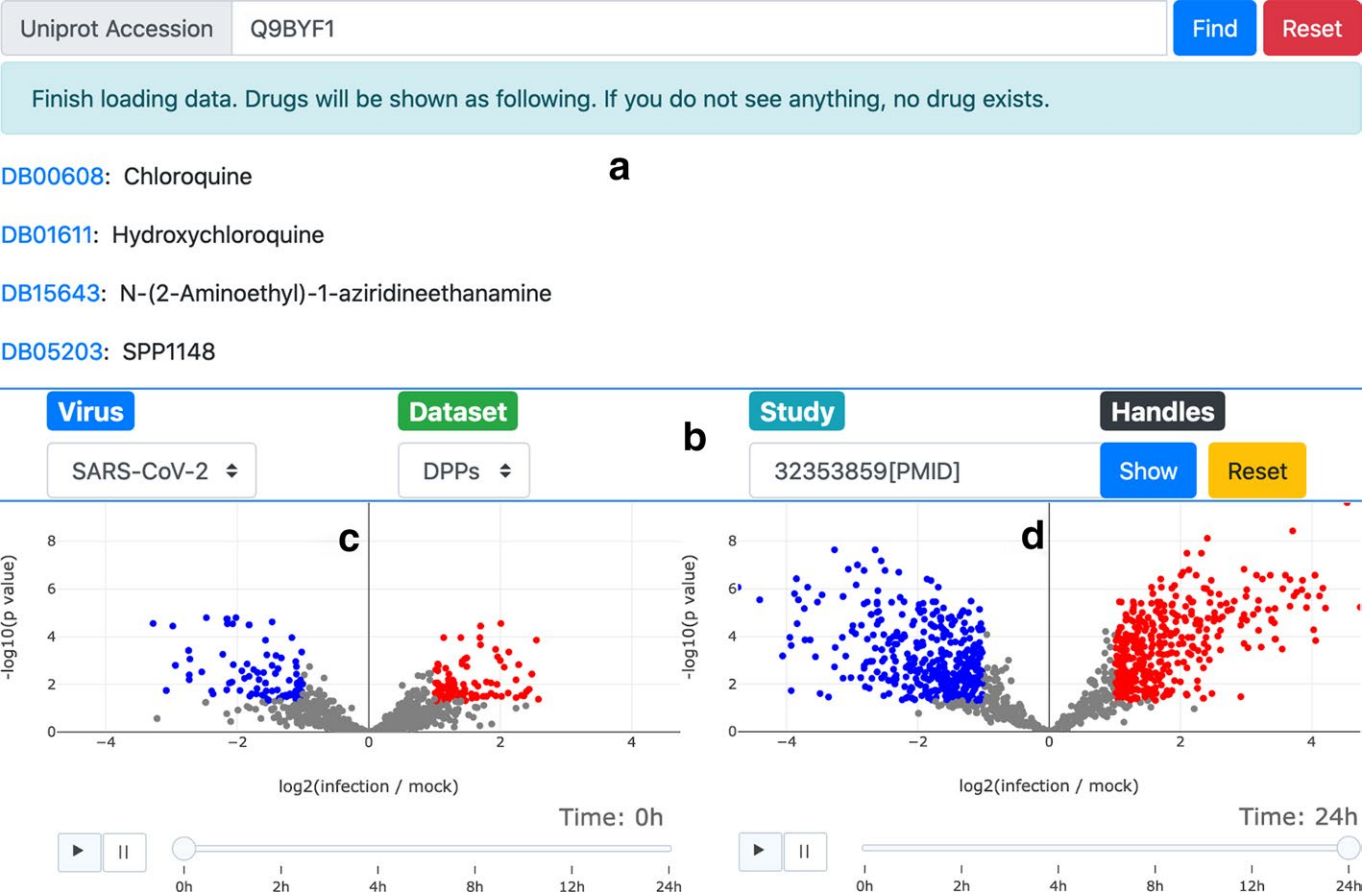
Utilities and examples. **a** Drug finder. **b** Settings panel for data animation. **c**, **d** Phosphorylation profile of all proteins at the beginning (**c**) and end (**d**). Of note, the animation can be played on the web page. For (**c**) and (**d**), the x-axis shows the log2-transformed fold change, the y-axis shows the negation of the log10-transformed p value, the blue points denote proteins with downregulated phosphorylation upon infection, the red points denote proteins with upregulated phosphorylation upon infection, and the gray points denote proteins without significant changes in phosphorylation upon infection

To facilitate rapid drug discovery for the treatment of COVID-19 during the pandemic, H2V provides a drug finder that can be used to identify drugs that target a given protein based on the UniProt accession number. The found drugs and their DrugBank identifiers will then be displayed on the lower part of the same page. For example, a search for Q9BYF1 will identify a few drugs, including chloroquine and hydroxychloroquine (Fig. 3a).

To help users establish a concrete perception of how all genes/proteins change dynamically over time post infection, H2V provides a utility called “Data animation”. On the page, a settings panel is provided to select data for animation. For example, Fig. 3b shows the setting to animate DPPs in response to SARS-CoV-2 infection. The results (Fig. 3c, d) of this example demonstrate that more human proteins are differentially phosphorylated at 24 h than immediately after SARS-CoV-2 infection. This indicates that the human body responds to SARS-CoV-2 infection by continuously rewiring cellular pathways.

**Fig. 4:**
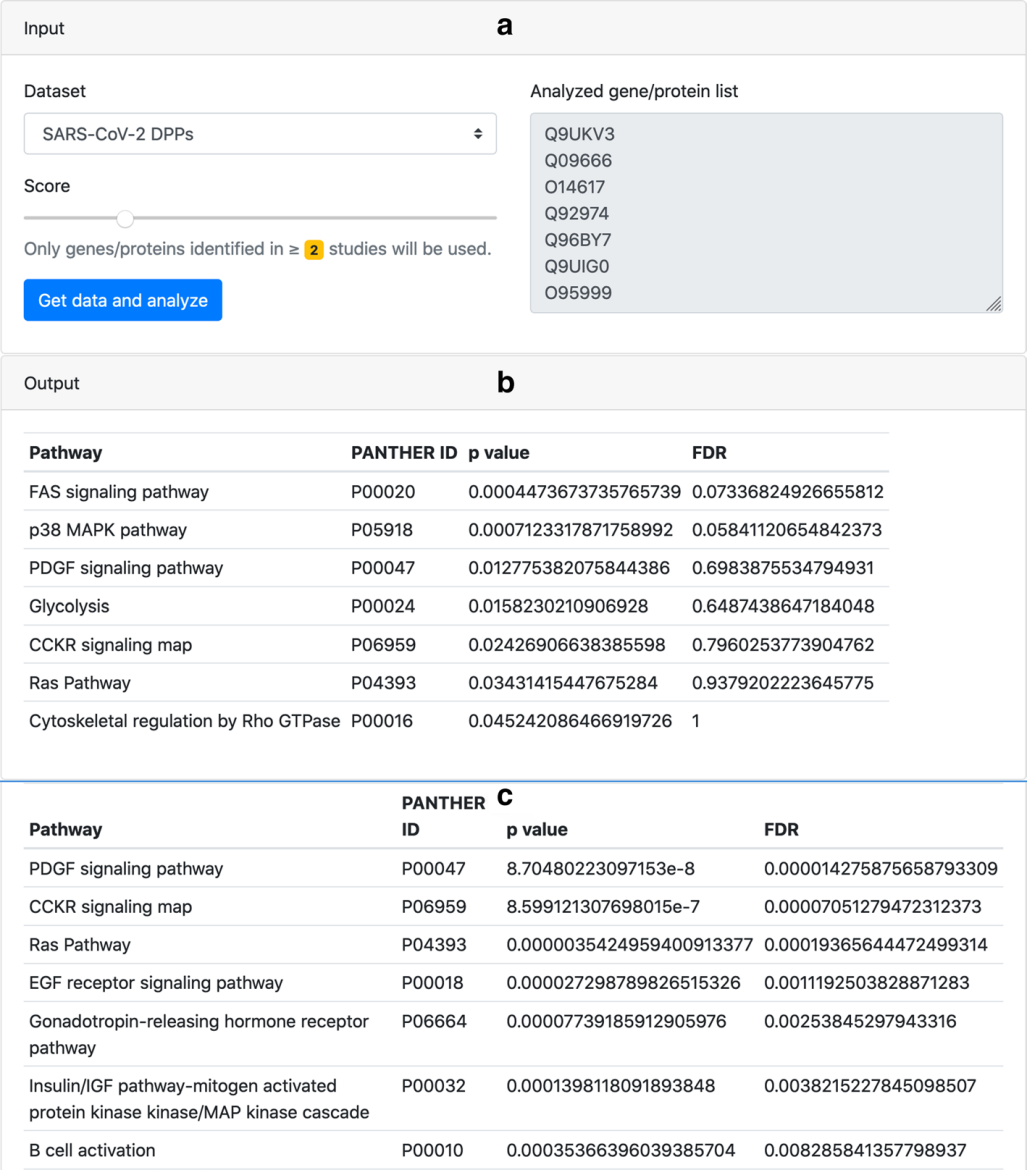
Enrichment analysis. **a** Input panel. **b** Pathway enrichment for DPPs identified in at least two studies. **c** Pathway enrichment for DPPs identified in at least one study (only the top seven pathways are shown)

H2V can be used to analyze integrated findings from different studies. Figure 4 shows an example of using the “Enrichment” analysis utility to analyze enriched pathways of DPPs that respond to SARS-CoV-2 infection. DPPs identified in at least two studies were analyzed first (also referred to as analysis 1). After setting the parameters on the left in Fig. 4a, the analysis was implemented by clicking the button at the bottom. Based on the completed analysis, the input DPPs for analysis are listed on the right in Fig. 4a, and the result is shown in Fig. 4b. Seven pathways were enriched, including the FAS signaling pathway, p38 MAPK pathway, and PDGF signaling pathway. Findings repeated in independent studies are expected to be more reliable than those from a single study, so the same analysis (referred to as analysis 2) was performed for DPPs identified in at least one study. This time, more pathways were enriched, and the top seven pathways are shown in Fig. 4c. The comparison shows that the top two pathways identified in analysis 1 were not among the top seven pathways identified in analysis 2. This indicates that the inclusion of DPPs of low confidence could distort the analysis result. H2V can be used to remove confounding factors to acquire reliable biological inferences.

## Conclusions

We have developed H2V as the first database of human proteins and genes that respond to SARS-CoV-2, SARS-CoV, and MERS-CoV infection. The database will help to understand the cellular details of how the human body responds to coronavirus infections. H2V can also be used as a platform to analyze rewired pathways by combining the findings from independent studies. This can be helpful to identify key targets with potential to treat coronavirus diseases. We acknowledge that the present release of our database may omit some data that should be included, and we will continue to update the database and provide the missing data in future releases. In summary, the database will help to design effective and specific therapeutics and preventive vaccines targeting SARS-CoV-2, SARS-CoV and MERS-CoV.

## Data Availability

All data generated or analyzed during this study are included in this published article.

## Abbreviations

SARS-CoV-2: Severe acute respiratory syndrome coronavirus 2
SARS-CoV: Severe acute respiratory syndrome coronavirus
MERS-CoV: Middle East respiratory syndrome-related coronavirus
COVID-19: Coronavirus disease 2019
WHO: World Health Organization
ACE2: Angiotensin-converting enzyme 2
DPP4: Dipeptidyl peptidase 4
DEGs: Differentially expressed genes
PPIs: Protein–protein interactions
DEPs: Differentially expressed proteins
DPPs: Differentially phosphorylated proteins
DTPs: Differentially translated proteins
SAPs: Severity associated proteins
NCBI: National center for biotechnology information
HTML: Hypertext markup language
CSS: Cascading style sheets
REST: Representational state transfer
API: Application programming interface
